# Assessing buprenorphine treatment utilization and SAMHSA DATA waiver provider distribution in 2021: a real-world analysis in California

**DOI:** 10.1038/s41598-025-07315-9

**Published:** 2025-08-09

**Authors:** Yun Wang, Tao Hu, Chih-Hung Chang, Matthew Heshmatipour, Jeff Jianfei Guo, Moom R. Roosan, Karen A. Miotto

**Affiliations:** 1 School of Pharmacy, Chapman University, 9401 Jeronimo Road, Irvine, CA 92618 USA; 2 Department of Geography, Oklahoma State University, Stillwater, USA; 3 Program in Occupational Therapy, Department of Medicine, Department of Orthopaedic Surgery, Washington University in St Louis School of Medicine, St. Louis, MO USA; 4 Department of Clinical Pharmacy Practice, School of Pharmacy & Pharmaceutical Sciences, University of California, Irvine, USA; 5 University of Cincinnati College of Pharmacy, University of Cincinnati Medical Center, Cincinnati, USA; 6 Department of Psychiatry and Biobehavioral Sciences, David Geffen School of Medicine, University of California, Los Angeles, CA USA

**Keywords:** Buprenorphine, DATA-waiver, Opioid use disorder, Medication for opioid use disorder, Geospatial, Health disparity, Ecological epidemiology, Epidemiology, Outcomes research

## Abstract

Qualified clinicians previously required a practitioner waiver (i.e., “DATA-waiver” or "X-waiver") to offer buprenorphine, a medication for opioid use disorder (OUD). However, many counties in the US experience fewer buprenorphine prescriptions due to factors such as “DATA-waiver” providers underutilizing their buprenorphine prescribing ability. Our study aimed to compare the availability of active buprenorphine-prescribing clinicians in California to the Substance Abuse and Mental Health Services Administration (SAMSHA)-listed DATA waived prescribers under each 5-digit ZIP Code. This study utilized the buprenorphine prescription record data in 2021 from California’s Controlled Substance Utilization Review and Evaluation System (CURES). The list of the locators for all DATA-waiver clinicians was obtained from the SAMHSA webpage. Among 1,600 ZIP Codes where patients resided, 62.1% housed DATA-waived physicians, and 57.8% had active buprenorphine-prescribing clinicians. A disproportional distribution between buprenorphine-prescribing clinicians and DATA-waived physicians was evident. Among physicians listed in the SAMHSA roster, not all were actively prescribing buprenorphine. Significant disparities in access to active prescribers and DATA waiver prescribers persist between rural and urban areas in California. Addressing these issues requires resource allocation and inter-professional collaboration. The 2022 Omnibus bill’s policy changes hold promise, necessitating further effort into OUD-based policy changes.

**Supplementary Information:**

The online version contains supplementary material available at 10.1038/s41598-025-07315-9.

## Introduction

The opioid epidemic in the United States has cast a dark shadow, resulting in a distressing surge in fatalities. In the battle against opioid use disorder (OUD), medications for opioid use disorder (MOUD) have emerged as the bedrock solution. Within the realm of MOUD, methadone and buprenorphine have been substantiated by a wealth of evidence as first-line treatments of OUD. However, the prescribing landscape for methadone presents a challenge due to stringent federal regulations mandating dispensation exclusively through authorized clinics and in liquid form ^1,2^. On the other hand, buprenorphine boasts several pharmacokinetic advantages over methadone, making it a compelling choice for use in primary care settings and as take-home medications ^1^. Buprenorphine, a partial mu-opioid receptor agonist, exhibits a “ceiling effect,” reducing respiratory depression and overdose risk compared to methadone, a full agonist with higher potency ^3^. It has an extended half-life of 24–60 hours^4^, thus supporting less frequent dosing while effectively managing withdrawal and cravings ^5^. Unlike methadone, which is associated with metabolic disturbances and requires daily administration at opioid treatment programs, buprenorphine has a safer profile, fewer drug interactions, and can be prescribed in community settings, enhancing accessibility and adherence. The Drug Addiction Treatment Act (DATA) of 2000 ^6^ marked a pivotal moment by allowing qualified physicians to obtain a waiver, commonly referred to as a “DATA waiver,” enabling them to prescribe buprenorphine medications for treating OUD. Despite this progressive step, studies have shown that many DATA-waivered physicians were not inclined to prescribe buprenorphine^1^. Astonishingly, roughly 40% of appointment requests from buprenorphine patients were denied by prescribers ^7^. In light of these challenges, our California research aimed to shed light on the percentage of DATA-waived clinicians actively prescribing buprenorphine within each 5-digit ZIP Code. Leveraging California’s prescription drug monitoring program (PDMP) data, our study seeks to quantify the gaps in accessing buprenorphine.

## Methods

Our study harnessed real-world data sourced from California’s Controlled Substance Utilization Review and Evaluation System (CURES), a state-operated PDMP database that documents the dispensation of Schedule II-V prescription drugs by outpatient pharmacies in California. Buprenorphine, categorized as a controlled Schedule III drug, is comprehensively included in the CURES database. We extracted all buprenorphine prescription records from California’s CURES database from January 1 to December 31, 2021. These records encompassed variables, including the patient’s city, state, and 5-digit ZIP code of residence, as well as the 5-digit ZIP code of both the prescriber and the dispensing pharmacy, product name, National Drug Code (NDC), form, strength, and quantity of the prescribed drug. Our data collection from CURES enabled us to identify the active buprenorphine-prescribing clinicians and patients who were buprenorphine recipients within each ZIP Code in California, utilizing unique prescriber and patient identifiers. We then procured an exhaustive roster of “DATA waived” clinicians, including their 5-digit ZIP Codes, counties, and practice addresses, from the Substance Abuse and Mental Health Services Administration (SAMHSA) Behavioral Health Treatment Services Locator database ^8^. In the final phase, we linked these two datasets by the 5-digit ZIP code, yielding a final dataset that encompasses the corresponding county/city names for each ZIP Code and the counts of patients receiving buprenorphine, active buprenorphine-prescribing clinicians, and DATA-waived clinicians operating within each ZIP Code.

### Statistical analysis

Descriptive analysis and gradient maps were employed to delineate the spatial distribution of ZIP Codes hosting DATA-waived clinicians and active prescribers, categorized by patient volumes. To categorize communities, our study employed the United States Department of Agriculture’s Rural-Urban Commuting Area Codes (RUCAC), encompassing classifications for metropolitan, micropolitan, small-town, and rural areas. We pictured the spatial relationship of DATA-waived clinicians, active buprenorphine prescribers, and patient volume under each ZIP code in nine class bivariate choropleth maps. A bivariate choropleth map ^9,10^ is a thematic map that displays two variables on a single map by combining two different sets of colors. For skewed variables such as the number of patients, prescribers, and DATA-waived practitioners we applied the natural breaks (Jenks) classification method to optimize group differentiation and enhance visual interpretability. As a result, the number of Prescribers was classified into three groups: low (1–17), medium (18–76), and high (77–186). Similarly, the number of Patients was grouped as low (1–16), medium (17–61), and high (62–678). For the number of DATA waiver practitioner, the classification included low (1–2), medium (3–6), and high (7–136). Consequently, the bivariate choropleth maps illustrated the relationships between these groups, focusing on two selected variables from a spatial perspective, as detailed in the figures. We employed multilinear regression models in supplementary tables to evaluate the association between patient volume, the number of DATA-waived clinicians, and active prescribers under each Zip Code with demographic and socio-economic attributes derived from the US Zip Codes Database (Pareto Software™, version 2023). We initially applied the Least Absolute Shrinkage and Selection Operator (LASSO) regression to mitigate multicollinearity and enhance variable selection in modeling the outcome variables. LASSO ^11^ is a regularization technique that introduces an *L1* penalty term to the objective function, effectively constraining the sum of absolute regression coefficients. This approach facilitates automatic variable selection by driving the coefficients of less informative predictors to zero, thereby retaining only the most influential covariates in the model. LASSO is particularly effective in handling high-dimensional data and reducing multicollinearity, although it may not completely eliminate severe multicollinearity ^12^. To further assess multicollinearity, we computed the Variance Inflation Factor (VIF) for all independent variables. Covariates exceeding a predefined VIF threshold of 5 ^13^ were either excluded or consolidated to improve model stability, robustness, and interpretability 37. A significance level of *p* < 0.05 was employed in the multivariate regression analyses to determine statistical significance. The statistical analyses were performed using Stata (version 17.0, StataCorp LLC, College Station, TX), while spatial analyses were conducted in ArcGIS Pro (version 2.9). The Institutional Review Board at Chapman University granted an exemption to review the study, acknowledging the deidentified nature of the dataset. Map created by authors using public shapefiles in ArcGIS. No copyrighted material was used.

## Results

**Fig. 1:**
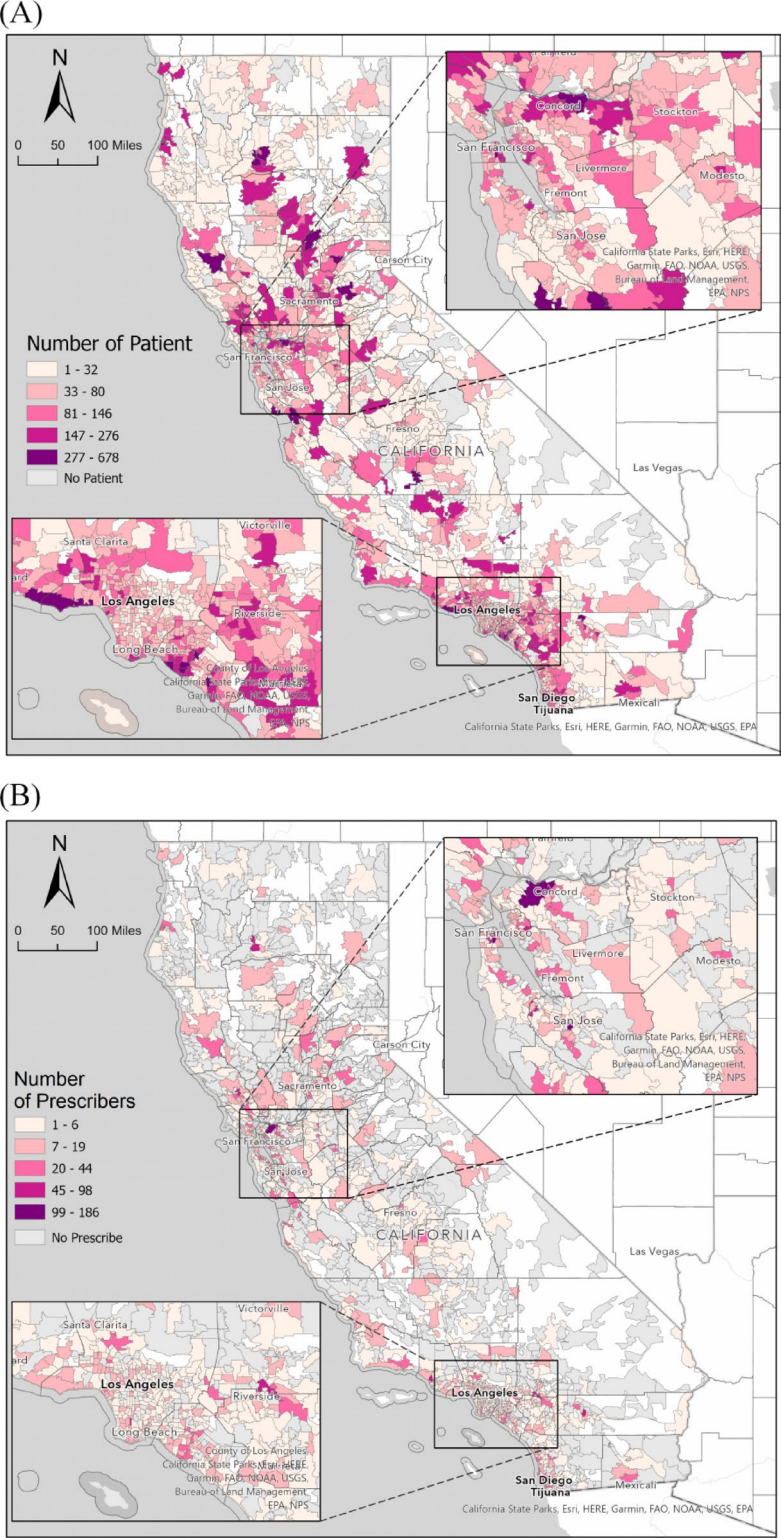

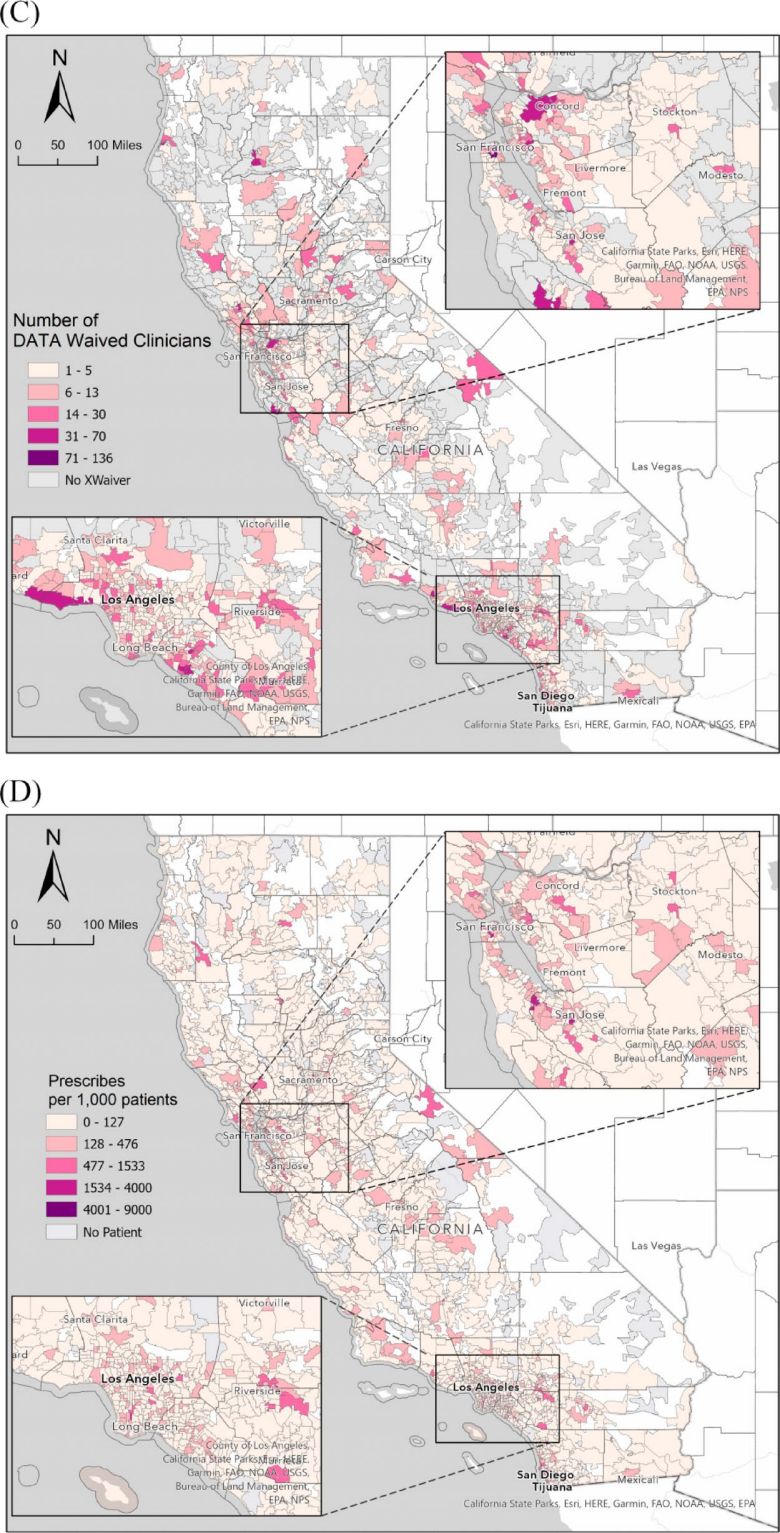

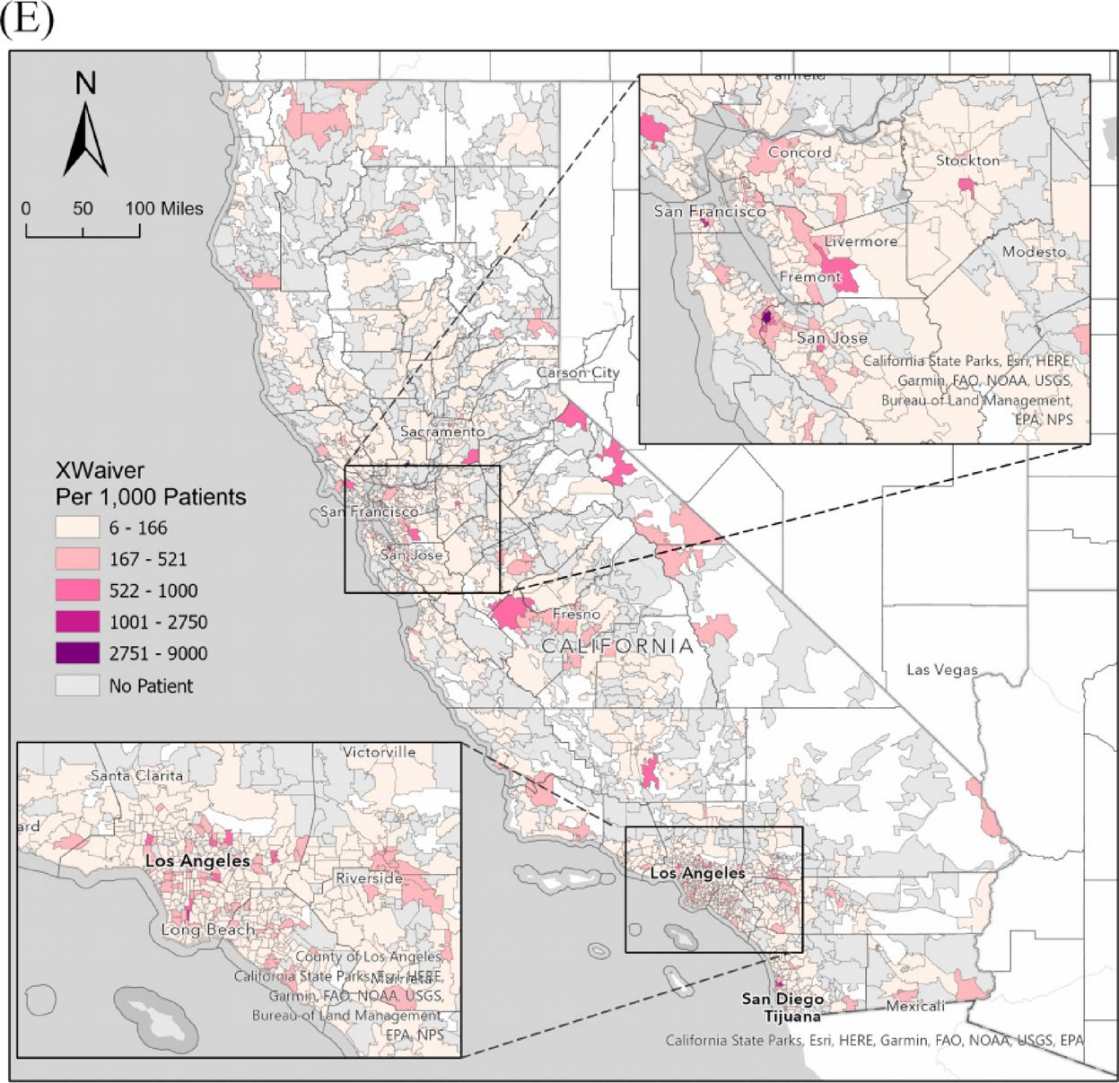
Gradient maps for the distribution of (**A**) patient volume, (**B**) active buprenorphine prescribers, (**C**) DATA waived clinicians, (**D**) prescribers per 1000 patients, and (**E**) DATA waiver providers per 1000 patients in California, 2021.

Between January and December 2021, 88,202 patients with buprenorphine prescriptions were recorded across 1,600 ZIP codes in the CURES. Of these ZIP Codes, 62.1% had 8,013 actively practicing buprenorphine-prescribing clinicians, and 57.8% had 6,699 DATA waiver-certified physicians across 925 ZIP codes. Our results from California reveal that 86% of patients reside in metropolitan regions, with only 1.6% in rural settings. A disproportionate 92% of DATA waivers and 92% of active prescribers are concentrated in metropolitan areas, with just 0.93% and 0.26% in rural areas, respectively (Fig. 1).

However, the distribution of these services was not uniform, with significantly higher concentrations in urban centers such as the Bay Area, Los Angeles, and San Diego, as highlighted in Fig. 1. ZIP codes in urban areas with higher population densities or major medical centers include 92103 (San Diego, covering Hillcrest and Mission Hills), 94110 (San Francisco, primarily the Mission District), 95128 (San Jose, around Valley Medical Center and parts of the West San Carlos neighborhood), 94553 (Martinez, the county seat of Contra Costa County), 90033 (Los Angeles, including Boyle Heights and the area around LAC + USC Medical Center), 95817 (Sacramento, home to the UC Davis Medical Center), 95128 (San Jose, near Valley Medical Center, a large public hospital), and 90033 (Los Angeles, location of LAC + USC Medical Center). These ZIP codes had the largest numbers of active buprenorphine prescribers (Fig. 1).

Our analysis reveals that regions with higher proportions of married individuals exhibit a lower density of DATA-waived prescribers, suggesting potential socio-cultural or economic barriers to provider availability (Supplementary Table 1). Population density emerges as a key determinant, with densely populated areas hosting a greater number of DATA-waived prescribers, reinforcing the role of urbanization in service distribution. Additionally, communities with higher proportions of Asian residents and elevated home ownership rates demonstrate lower provider availability. Areas with higher uninsured rates also show reduced prescriber presence. Similarly, active buprenorphine prescribers are more prevalent in regions with greater housing availability but remain less common in areas characterized by higher proportions of married individuals and larger family sizes. Areas with higher Black populations exhibit fewer active prescribers, underscoring potential disparities in treatment access.

A few ZIP codes had active patients but no registered SAMHSA DATA-waiver providers, including 92585 (Sun City, growing community with new housing developments), 90303 (Inglewood, diverse community with a significant African American population), 96007 (Anderson, rural area), 95003 (Aptos, rural area), 95,380 (Turlock, agricultural hub), 93,023 (Ojai, relatively small city), 95713 (Colfax, recreational town), 95552 (Korbel, outdoor recreational town), 95842 (Sacramento, suburban area), 93444 (Nipomo, suburban area), 91,306 (Winnetka, residential neighborhood), 93505 (California City, focus on renewable energy and aerospace industries), and 94933 (Forest Knolls, rural community with a focus on natural beauty and outdoor activities). This distribution highlights the disparity in access to buprenorphine-prescribing clinicians and DATA waiver-certified physicians, with certain urban areas demonstrating a significantly higher provision of services than rural and suburban regions.

**Fig. 2:**
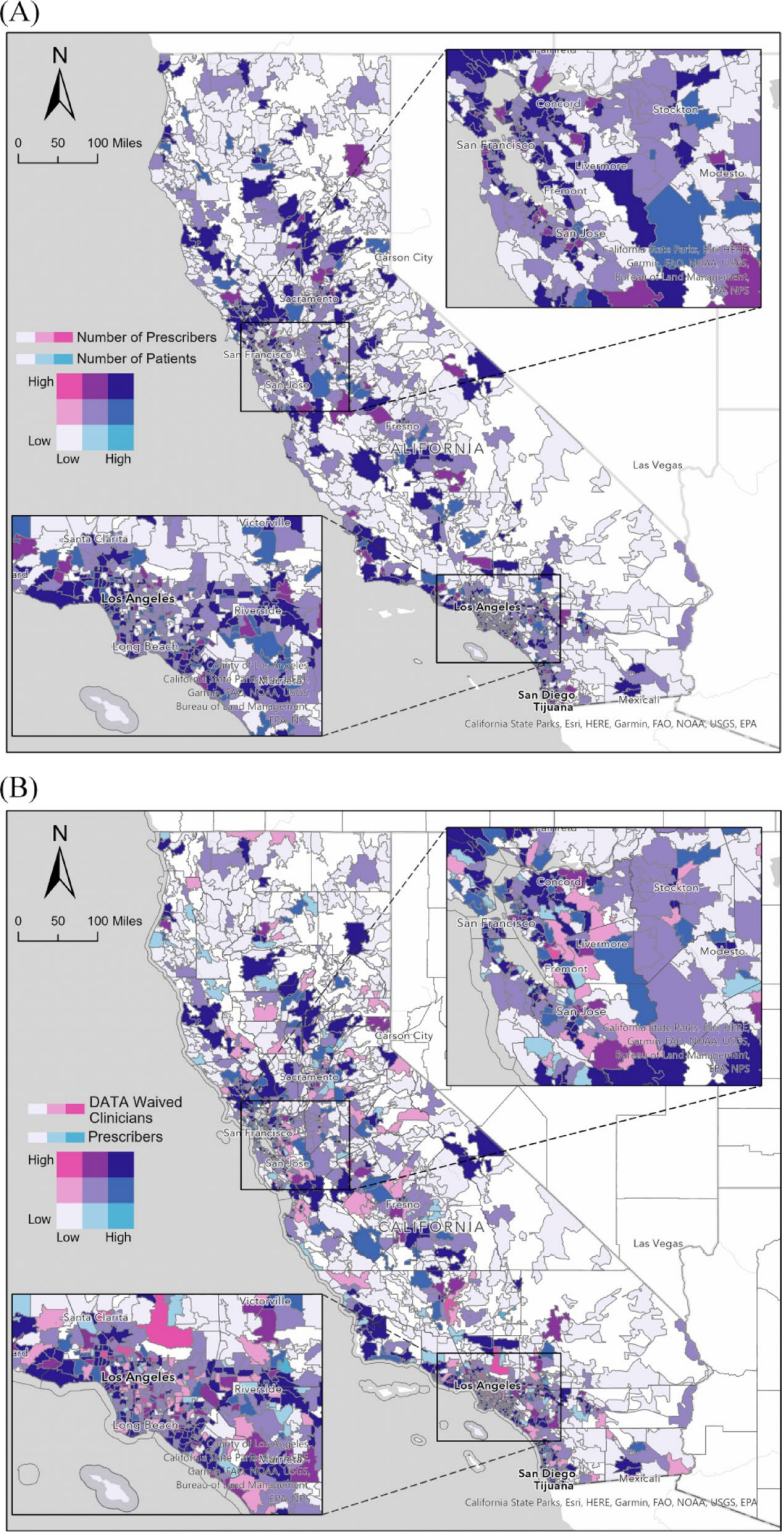

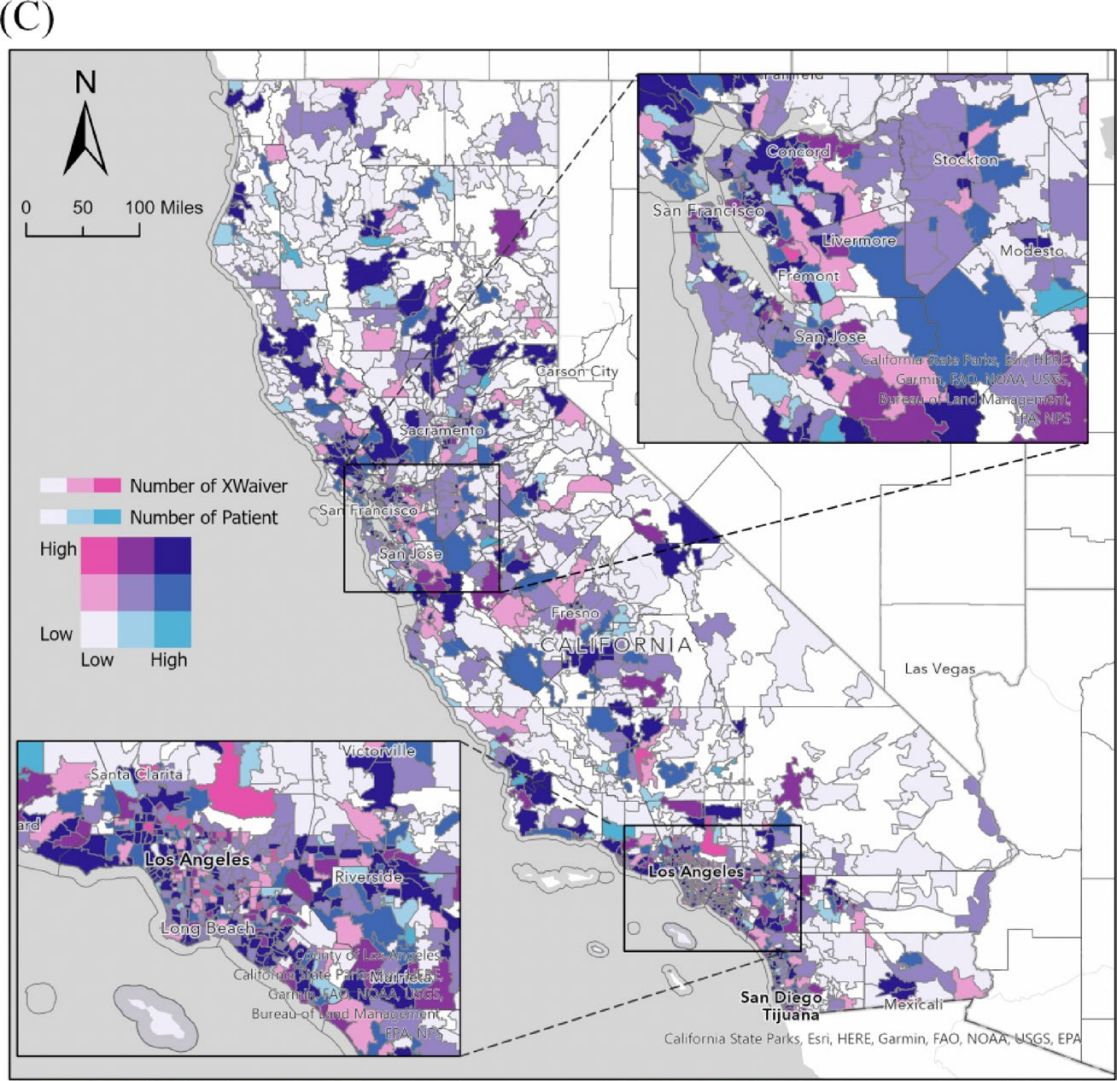
Nine-class bivariate choropleth map of (**A**) distributions active buprenorphine prescribers and patient volume, (**B**) distributions of DATA-waived clinicians and active buprenorphine prescribers, and (**C**) distributions of DATA-waived clinicians and patient volume in California, 2021.

Figure 2 (A) provides a detailed analysis of the relationship between active buprenorphine prescribers and patients across California. The map reveals a generally well-distributed pattern, with prescribers aligning with patient numbers in most communities. No communities exhibit significant imbalances, such as an excessive number of patients with a shortage of prescribers or a surplus of prescribers with a limited patient population. Figure 2(B) illustrates the relationship between active prescribers and clinicians holding DATA waivers. Certain regions, particularly those near Riverside and northern California, still face a shortage of these waived clinicians. Unlike the balanced distribution between prescribers and patients in Fig. 2(A), 20 ZIP Codes in Fig. 2(B) expose spatial disparities between DATA waivers and active prescribers. Remarkably, 16 ZIP codes display a notable abundance of DATA-waived clinicians but a deficiency of prescribers, primarily clustered in the Los Angeles area, indicated by dark pink in Fig. 2(B). Conversely, 4 ZIP codes exhibit a surplus of prescribers compared to the number of DATA-waived clinicians, as indicated by cyan in Fig. 2(B). This underscores the unique distribution between DATA-waived clinicians and buprenorphine prescribers.

## Discussion

Our comprehensive analysis of buprenorphine-prescribing clinicians and DATA-waived physicians across California in 2021 provides critical insights into the structural and systemic barriers shaping healthcare accessibility and provider engagement in OUD treatment. Consistent with extant literature ^14^, our findings underscored a pronounced disjunction between regulatory compliance, as reflected in waiver attainment, and actual prescribing behavior. A substantial proportion of DATA-waived providers were not actively prescribing buprenorphine despite voluntarily registering with the SAMHSA Treatment Locator—a resource intended to enhance patient access by signaling provider availability ^15^. This discrepancy suggests that regulatory authorization alone cannot translate into clinical practice, implicating entrenched barriers beyond administrative prerequisites. Persistent geographic disparities, particularly in rural and suburban regions, continue to impede equitable access to OUD pharmacotherapy. While we acknowledge the recent policy revision eliminating the DATA waiver requirement for buprenorphine prescribing, a rigorous evaluation of prescribing trends among SAMHSA-listed (formerly DATA-waived) providers remains essential for multiple reasons. Removing a regulatory hurdle does not precipitate immediate shifts in clinical practice. Prior waiver recipients likely possess a higher degree of familiarity with buprenorphine treatment protocols, rendering them more inclined to incorporate MOUD into their clinical practice . Historical prescribing patterns among SAMHSA-listed providers constitute a critical baseline for assessing the policy change’s efficacy. Even without a formal waiver requirement, many previously DATA-waived providers likely underwent specialized training or demonstrated a sustained commitment to OUD treatment, positioning them as a pivotal cohort for targeted interventions. A nuanced understanding of these provider dynamics is imperative for informing evidence-based educational initiatives and policy frameworks designed to optimize buprenorphine prescribing capacity.

Our findings reveal a pronounced urban concentration of active buprenorphine prescribers, with dense provider clusters in metropolitan centers such as the Bay Area, Los Angeles, and San Diego. This distribution reinforces the role of population density as a determinant of healthcare accessibility, wherein urban settings benefit from higher provider availability and robust treatment infrastructure. In stark contrast, rural regions remain markedly underserved. While nearly two-thirds of rural counties contain at least one DEA-waived clinician, over half of small and remote rural counties lack any active buprenorphine prescriber. The compounded effects of geographic isolation, workforce shortages, and persistent stigma surrounding OUD treatment in close-knit communities exacerbate access inequities. Certain suburban and rural areas, such as Redding and El Centro, demonstrate sustained prescriber engagement, but these instances remain anomalous within the broader context of provider shortages. Furthermore, our analysis suggests that while California exhibits an overall alignment between prescribers and patient volume, notable regional disparities exist. For instance, in Los Angeles, specific areas demonstrate an oversupply of DATA-waived clinicians but a shortage of active prescribers, while others display overabundance of DATA-waived clinicians but a shortage of prescribers.

A closer analysis of the geographical distribution of active prescribers and clinicians with DATA waivers offered insights into the complexities of service provision. California demonstrated a general alignment between prescriber availability and patient volume at the state level; however, notable regional disparities in access remain, particularly in rural and underserved areas. However, our findings uncovered disparities, particularly in Los Angeles, where certain areas have an overabundance of DATA-waived clinicians but a shortage of prescribers, while others exhibit the reverse scenario. This observation raised questions about the effective coordination and communication among healthcare providers in optimizing service distribution.

Our study systematically examines the intersection of socio-demographic determinants and the geographic distribution of buprenorphine prescribers, elucidating structural disparities in OUD treatment access. Prior research has documented disproportionately high buprenorphine prescription refusal rates among African Americans ^16^, indicating systemic barriers to care. Our analysis revealed a lower density of active prescribers in areas with higher Black populations. Regions with higher proportions of married individuals exhibit lower prescriber densities. Urbanization emerges as a key factor, with densely populated areas hosting more prescribers, whereas regions with higher proportions of Asian residents and elevated homeownership rates exhibit reduced provider presence. Longer commute times correlate with lower prescriber density, emphasizing the role of transportation in healthcare access. Additionally, areas with higher uninsured rates exhibit fewer DATA-waived prescribers, reinforcing the economic constraints limiting OUD treatment availability in underinsured populations. Healthcare demand, as proxied by patient volume, correlates positively with housing unit density, suggesting a link between residential concentration and treatment utilization. Consistent with trends observed among DATA-waived prescribers, active buprenorphine providers are more prevalent in regions with greater housing availability but less common in areas with higher proportions of married individuals and larger family sizes.

Our study had several limitations. Our study’s data linkage relied solely on a 5-digit ZIP Code due to Health Insurance Portability and Accountability Act (HIPAA) constraints, ensuring individual anonymity. However, this approach may not accurately identify DATA-waived clinicians who are not actively engaged in prescribing. The imperative to enhance timely access to buprenorphine treatment for OUD through policy changes has long been acknowledged. The 2022 Omnibus bill represents a significant step forward by eliminating the federal mandate for practitioners to secure a DATA waiver before prescribing buprenorphine. This progressive shift aims to expand the reach of this effective treatment, enhance its availability, and ensure greater accessibility for individuals seeking OUD care. Our pre-2022 Omnibus bill investigation underscored a noticeable contrast between active buprenorphine prescribers and clinicians possessing DATA waivers, emphasizing the necessity for such progressive measures. However, we are unable to address the reasons why not all DATA-waived prescribers actively prescribed. The policy change to remove legislative and regulatory restrictions after the Omnibus bill might motivate prescribers to manage more patients. Further research is needed to evaluate the effectiveness of this policy change.

In conclusion, California has made significant strides in providing buprenorphine to patients in need. However, addressing regional disparities in access to these services is imperative. Future endeavors should prioritize strategically allocating resources to regions with a shortage of active prescribers.

## Electronic supplementary material

Below is the link to the electronic supplementary material.

Supplementary Material 1

## Data Availability

The dataset used in this study is available upon request from the California Department of Justice. For details on the data request process, please visit: https://oag.ca.gov/research-center/request-process. The point of contact from the author’s side is Dr. Yun Wang (yunwang@chapman.edu).
