# Supplementary material for: Assessing buprenorphine treatment utilization and SAMHSA DATA waiver provider distribution in 2021: a real-world analysis in California
Source: Sci Rep. 2025 Aug 9;15:29168. doi: 10.1038/s41598-025-07315-9 (PMC12335559; doi:10.1038/s41598-025-07315-9)
Supplement: Supplementary file 1 — Supplementary Material 1 [file 41598_2025_7315_MOESM1_ESM.docx]

**Supplementary Table 1: Statistically Significant ZIP Code Characteristics Associated with High Patient Volume, X-Waived Clinician Density, and Active Prescriber Count – A Multivariate Linear Regression Analysis**

| **ZIP Code based characteristics** | **Beta Coefficient** | **P value** | **(95% CI)** | |
| --- | --- | --- | --- | --- |
| **Outcome: Patient volume** | | | | |
| housing units | 0.007 | <0.001 | 0.006 | 0.007 |
| race: asian | -1.188 | <0.001 | -1.454 | -0.921 |
| labor force participation | -1.101 | <0.001 | -1.648 | -0.554 |
| dual income family | 0.605 | 0.003 | 0.202 | 1.009 |
| married | -0.773 | 0.003 | -1.289 | -0.256 |
| commute time | -0.947 | <0.001 | -1.366 | -0.528 |
| rural area | -14.325 | 0.005 | -24.218 | -4.431 |
| race: black | -0.710 | 0.002 | -1.165 | -0.255 |
| male | 1.384 | 0.002 | 0.524 | 2.245 |
| divorced | 1.090 | 0.017 | 0.192 | 1.989 |
| education: graduate | -0.543 | 0.024 | -1.016 | -0.070 |
| household income from $10,000-$15,000 | 1.152 | 0.036 | 0.078 | 2.226 |
| age: 40s | 1.263 | 0.012 | 0.279 | 2.247 |
| race: other | -0.722 | <0.001 | -1.029 | -0.415 |
| age: 30s | 1.128 | 0.004 | 0.366 | 1.890 |
| race: Native Hawaiian/Pacific Islander | 4.938 | 0.044 | 0.132 | 9.745 |
| race: multiple | -0.903 | 0.007 | -1.556 | -0.250 |
| **Outcome: Number of DATA waived clinicians** | | | | |
| married | -0.098 | 0.004 | -0.165 | -0.031 |
| population under this ZIP Code | 0.00011 | <0.001 | 0.00009 | 0.00014 |
| race: asian | -0.102 | <0.001 | -0.142 | -0.062 |
| home ownership: yes | -0.071 | <0.001 | -0.108 | -0.034 |
| population per square kilometer | 0.0004 | 0.003 | 0.0001 | 0.001 |
| commute time | -0.098 | 0.001 | -0.156 | -0.040 |
| uninsured | -0.182 | 0.003 | -0.303 | -0.060 |
| age: 10 to 19 | -0.161 | 0.006 | -0.277 | -0.046 |
| education: some college | -0.066 | 0.034 | -0.127 | -0.005 |
| **Outcome: Number of active prescribers** | | | | |
| housing units | 0.0005 | <0.001 | 0.0004 | 0.0006 |
| married | -0.144 | 0.001 | -0.225 | -0.063 |
| race:asian | -0.097 | <0.001 | -0.151 | -0.044 |
| family size | -2.051 | 0.013 | -3.666 | -0.436 |
| commute time | -0.100 | 0.019 | -0.183 | -0.017 |
